# 5-Amino-6-benzoyl-8-nitro-2,3-di­hydro-1*H*-spiro­[imidazo[1,2-*a*]pyridine-7,3′-indolin]-2′-one dimethyl sulfoxide monosolvate

**DOI:** 10.1107/S1600536814008800

**Published:** 2014-04-26

**Authors:** R. A. Nagalakshmi, J. Suresh, S. Sivakumar, R. Ranjith Kumar, P. L. Nilantha Lakshman

**Affiliations:** a Department of Physics, The Madura College, Madurai 625 011, India; b Department of Organic Chemistry, School of Chemistry, Madurai Kamaraj University, Madurai 625 021, India; c Department of Food Science and Technology, University of Ruhuna, Mapalana, Kamburupitiya 81100, Sri Lanka

## Abstract

In the title compound C_21_H_17_N_5_O_4_·C_2_H_6_OS, the central six-membered ring derived from 1,4-di­hydro­pyridine adopts a distorted boat conformation with a small puckering amplitude of 0.127 (3) Å. The sums of bond angles around the pyridine N atom [358.7 (2)°] and the other imidazolidine N atom [60 (2)°] indicate that these atoms are in *sp^2^* hybridization, leading to an essentially planar imidazolidine ring. The last heterocycle, an oxindole moiety, is also nearly planar with an r.m.s. deviation of 0.0185 (1) Å. The amine NH_2_ group forms an intra­molecular hydrogen bond with the benzoyl group, giving a *S*(6) motif. In the crystal, N—H⋯O hydrogen bonds lead to the formation of chains along the *c*-axis direction. Within the chains there are further N—H⋯O and C—H⋯O hydrogen bonds enclosing *R* ^2^_2_(14) ring motifs. The chains are linked *via* N—H⋯O and C—H⋯O hydrogen bonds involving the dimethyl sulfoxide solvent mol­ecule which acts as both an acceptor and a donor..

## Related literature   

For a previous related work, see: Suresh *et al.* (2013 ▶). For conformational analysis of ring systems, and small rings fused to benzene, see: Cremer & Pople (1975 ▶); Allen (1981 ▶).
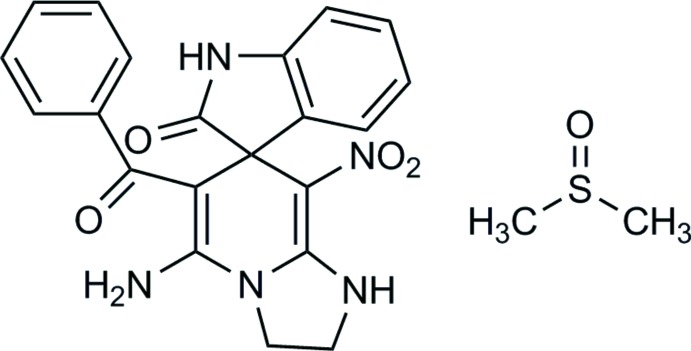


## Experimental   

### 

#### Crystal data  

C_21_H_17_N_5_O_4_·C_2_H_6_OS
*M* *_r_* = 481.52
Monoclinic, 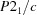

*a* = 16.476 (3) Å
*b* = 13.527 (2) Å
*c* = 10.0727 (18) Å
β = 99.868 (5)°
*V* = 2211.7 (7) Å^3^
*Z* = 4
Mo *K*α radiation
μ = 0.19 mm^−1^
*T* = 293 K
0.21 × 0.19 × 0.18 mm

#### Data collection  

Bruker Kappa APEXII diffractometer
Absorption correction: multi-scan (*SADABS*; Bruker, 2004 ▶) *T*_min_ = 0.967, *T*_max_ = 0.974
31940 measured reflections
4109 independent reflections
2818 reflections with *I* > 2σ(*I*)
*R*_int_ = 0.070

#### Refinement  

*R*[*F* ^2^ > 2σ(*F* ^2^)] = 0.056
*wR*(*F* ^2^) = 0.160
*S* = 1.05
4109 reflections
307 parameters
H-atom parameters constrained
Δρ_max_ = 0.92 e Å^−3^
Δρ_min_ = −0.47 e Å^−3^

### 

Data collection: *APEX2* (Bruker, 2004 ▶); cell refinement: *SAINT* (Bruker, 2004 ▶); data reduction: *SAINT*; program(s) used to solve structure: *SHELXS97* (Sheldrick, 2008 ▶); program(s) used to refine structure: *SHELXL97* (Sheldrick, 2008 ▶); molecular graphics: *PLATON* (Spek, 2009 ▶); software used to prepare material for publication: *SHELXL97*.

## Supplementary Material

Crystal structure: contains datablock(s) global, I. DOI: 10.1107/S1600536814008800/bh2496sup1.cif

Structure factors: contains datablock(s) I. DOI: 10.1107/S1600536814008800/bh2496Isup2.hkl

CCDC reference: 998123

Additional supporting information:  crystallographic information; 3D view; checkCIF report

## Figures and Tables

**Table 1:** Hydrogen-bond geometry (Å, °)

*D*—H⋯*A*	*D*—H	H⋯*A*	*D*⋯*A*	*D*—H⋯*A*
N2—H2*B*⋯O4	0.86	1.92	2.549 (3)	129
N2—H2*A*⋯O3^i^	0.86	2.30	2.939 (3)	131
N3—H3⋯O5^ii^	0.86	1.92	2.779 (4)	177
N5—H5⋯O3^iii^	0.86	2.31	2.924 (3)	129
C6—H6*B*⋯O2^iii^	0.97	2.59	3.274 (4)	128
C11—H11⋯O4^i^	0.93	2.43	3.346 (4)	167
C15—H15*C*⋯O2^iv^	0.96	2.55	3.448 (4)	156

Symmetry codes: (i) 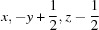; (ii) 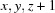; (iii) 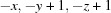; (iv) 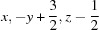.

**Fig. 1.**
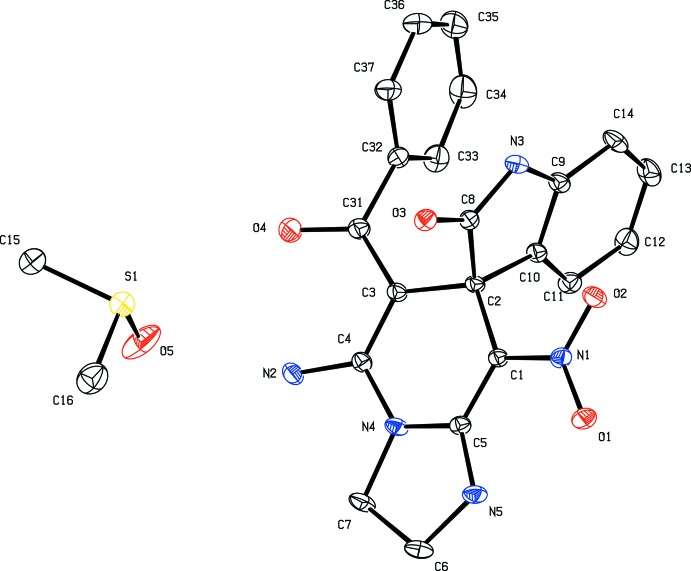
The molecular structure of the title compound, showing 20% probability displacement ellipsoids. All H atoms are omitted for clarity.

**Fig. 2.**
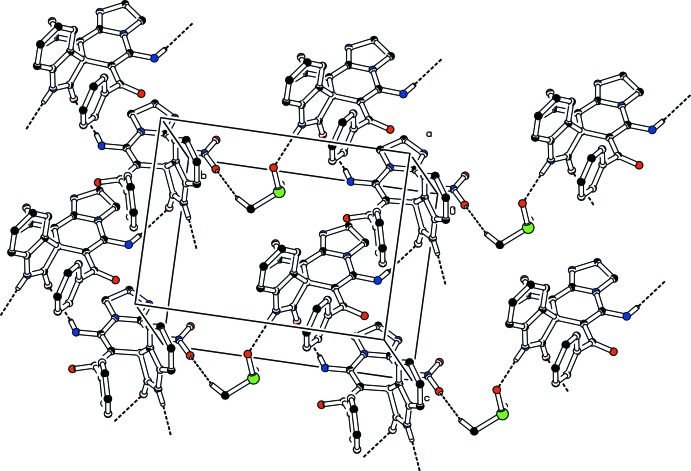
Partial packing diagram of the title compound. Dashed bonds represent inter-molecular hydrogen bonds.

## supplementary crystallographic information

### 2.1. Synthesis and crystallization

A mixture of benzoyl­aceto­nitrile (1.0 mmol), isatin (1.0 mmol) and 2-(nitro­methyl­ene) imidazolidine was dissolved in 10 ml of EtOH, and tri­ethyl­amine (1.0 mmol) was added. The reaction mixture was refluxed for 45 min. After completion of the reaction, as evident from TLC, the precipitated solid product was filtered and dried to obtain pure pale brown solid. Yield 94%. Melting point 530 K.

### 2.2. Refinement

Crystal data, data collection and structure refinement details are summarized in Table 1. H atoms were placed in calculated positions and allowed to ride on their carrier atoms with C—H = 0.93 (aromatic CH), 0.96 (methyl CH_3_) or 0.97 Å (methyl­ene CH_2_), and N—H = 0.86 Å. Isotropic displacement parameters for H atoms were calculated as *U*_iso_ = 1.5*U*_eq_(C) for CH_3_ groups and *U*_iso_ = 1.2*U*_eq_(carrier atom) for all other H atoms.

### 3. Results and discussion

Our inter­est in preparing pharmacologically active pyridine-related compounds (Suresh *et al.*, 2013) led us to the title compound, derived from a 1,4-di­hydro­pyridine, and we have undertaken X-ray crystal structure determination of this compound in order to establish its molecular conformation.

In the title compound (Fig. 1), the central pyridine ring adopts a skew-boat conformation with the puckering parameters *Q* = 0.127 (3) Å, θ = 87.3 (13) and φ = 198.3 (3)° (Cremer & Pople, 1975). The sums of bond angles around N4 and N5, 358.7 (2)° and 360 (2)° respectively, show that N atoms are in *sp*^2^ hybridization, leading to an essentially planar imidazolidine ring. The C2/C8/N3/C9/C10 ring of the oxindole moiety is planar with r.m.s. deviation of 0.0185 (1) Å. The small tilt between the planes of the five and six-membered rings in the oxindole unit is 1.21 (1)°. The sum of the bond angles around N3 atom is 360 (1)° implying a noticeable flattening of the geometry about N3. The shorter bond lengths N3—C8 = 1.337 (4) Å and N3—C9 = 1.401 (4) Å indicate the electron donating effect of the N atom. The nitro group, N1/O1/O2, is twisted away from the mean plane of the six-membered ring, forming the dihedral angle of 8.90 (1)°. In the benzene ring (C9···C14) of the oxindole ring system, the expansion of the *ipso* angles at C9, C12 and C13 [121.9 (3), 120.8 (3) and 121.3 (3)°, respectively] and the contraction of the apical angles at C10, C11 and C14 [120.5 (3), 118.1 (3) and 117.3 (3)°, respectively] are caused by the fusion of the smaller ring to the six-membered benzene ring, and the strain is taken up by the angular distortion rather than by bond length distortions (Allen, 1981). The short contacts H2A···H7A (2.17 Å) and H2A···H7B (2.31 Å) result in the substantial widening of angle C7—N4—C4 to 124.9 (2)°.

The crystal structure features weak intra-molecular N—H···O inter­actions and N—H···O and C—H···O inter-molecular inter­actions. An inter-molecular N2—H2A···O3 inter­action forms a chain along the *c* axis, while inter-molecular N5—H5···O3 and C6—H6B···O2 inter­actions form ring motifs *R*_2_^2^(14). The solvent molecule, dimetyl sulfoxide, also takes part in the N—H···O and in the C—H···O inter-molecular inter­actions (Fig. 2).

### Crystal data

**Table:**

C_21_H_17_N_5_O_4_·C_2_H_6_OS	*F*(000) = 1008
*M**_r_* = 481.52	*D*_x_ = 1.446 Mg m^−^^3^
Monoclinic, *P*2_1_/*c*	Melting point: 530 K
Hall symbol: -P 2ybc	Mo *K*α radiation, λ = 0.71073 Å
*a* = 16.476 (3) Å	Cell parameters from 2000 reflections
*b* = 13.527 (2) Å	θ = 2–31°
*c* = 10.0727 (18) Å	µ = 0.19 mm^−^^1^
β = 99.868 (5)°	*T* = 293 K
*V* = 2211.7 (7) Å^3^	Block, brown
*Z* = 4	0.21 × 0.19 × 0.18 mm

### Data collection

**Table:**

Bruker Kappa APEXII diffractometer	4109 independent reflections
Radiation source: fine-focus sealed tube	2818 reflections with *I* > 2σ(*I*)
Graphite monochromator	*R*_int_ = 0.070
Detector resolution: 0 pixels mm^-1^	θ_max_ = 25.5°, θ_min_ = 2.5°
ω and φ scans	*h* = −19→19
Absorption correction: multi-scan (*SADABS*; Bruker, 2004)	*k* = −16→16
*T*_min_ = 0.967, *T*_max_ = 0.974	*l* = −12→12
31940 measured reflections	

### Refinement

**Table:**

Refinement on *F*^2^	Primary atom site location: structure-invariant direct methods
Least-squares matrix: full	Secondary atom site location: difference Fourier map
*R*[*F*^2^ > 2σ(*F*^2^)] = 0.056	Hydrogen site location: inferred from neighbouring sites
*wR*(*F*^2^) = 0.160	H-atom parameters constrained
*S* = 1.05	*w* = 1/[σ^2^(*F*_o_^2^) + (0.0634*P*)^2^ + 2.4142*P*] where *P* = (*F*_o_^2^ + 2*F*_c_^2^)/3
4109 reflections	(Δ/σ)_max_ < 0.001
307 parameters	Δρ_max_ = 0.92 e Å^−^^3^
0 restraints	Δρ_min_ = −0.47 e Å^−^^3^
0 constraints	

### Fractional atomic coordinates and isotropic or equivalent isotropic displacement parameters (Å^2^)

**Table:**

	*x*	*y*	*z*	*U*_iso_*/*U*_eq_	
C1	0.12735 (16)	0.47401 (19)	0.4943 (3)	0.0273 (6)	
C2	0.19857 (15)	0.4409 (2)	0.6005 (3)	0.0268 (6)	
C3	0.20085 (16)	0.3272 (2)	0.6119 (3)	0.0285 (6)	
C4	0.14829 (16)	0.2694 (2)	0.5220 (3)	0.0292 (6)	
C5	0.08157 (16)	0.4092 (2)	0.4049 (3)	0.0292 (6)	
C6	−0.01200 (18)	0.3357 (2)	0.2370 (3)	0.0429 (8)	
H6A	−0.0032	0.3343	0.1442	0.051*	
H6B	−0.0705	0.3291	0.2384	0.051*	
C7	0.03642 (18)	0.2551 (2)	0.3186 (3)	0.0432 (8)	
H7A	0.0010	0.2147	0.3641	0.052*	
H7B	0.0641	0.2132	0.2622	0.052*	
C8	0.18947 (16)	0.4842 (2)	0.7405 (3)	0.0293 (6)	
C9	0.30975 (16)	0.5444 (2)	0.6929 (3)	0.0330 (7)	
C10	0.27937 (15)	0.4874 (2)	0.5812 (3)	0.0295 (6)	
C11	0.32187 (17)	0.4803 (2)	0.4754 (3)	0.0371 (7)	
H11	0.3022	0.4414	0.4006	0.045*	
C12	0.39483 (19)	0.5327 (2)	0.4833 (4)	0.0473 (8)	
H12	0.4244	0.5293	0.4127	0.057*	
C13	0.42409 (19)	0.5898 (3)	0.5948 (4)	0.0494 (9)	
H13	0.4731	0.6246	0.5978	0.059*	
C14	0.38202 (18)	0.5964 (2)	0.7022 (4)	0.0434 (8)	
H14	0.4019	0.6346	0.7775	0.052*	
C15	0.2595 (3)	0.6930 (3)	0.2703 (4)	0.0615 (10)	
H15A	0.3158	0.6979	0.3147	0.092*	
H15B	0.2246	0.6827	0.3362	0.092*	
H15C	0.2439	0.7531	0.2218	0.092*	
C16	0.1423 (2)	0.6058 (3)	0.0959 (4)	0.0730 (12)	
H16A	0.1245	0.5554	0.0302	0.109*	
H16B	0.1319	0.6697	0.0550	0.109*	
H16C	0.1126	0.5995	0.1695	0.109*	
C31	0.25828 (18)	0.2769 (2)	0.7134 (3)	0.0362 (7)	
C32	0.33727 (18)	0.3217 (2)	0.7853 (3)	0.0363 (7)	
C33	0.4043 (2)	0.3233 (3)	0.7190 (4)	0.0495 (9)	
H33	0.3980	0.3065	0.6283	0.059*	
C34	0.4805 (2)	0.3501 (3)	0.7889 (5)	0.0672 (12)	
H34	0.5259	0.3500	0.7453	0.081*	
C35	0.4901 (3)	0.3769 (3)	0.9213 (5)	0.0768 (14)	
H35	0.5418	0.3951	0.9672	0.092*	
C36	0.4236 (3)	0.3768 (3)	0.9867 (4)	0.0719 (13)	
H36	0.4299	0.3968	1.0763	0.086*	
C37	0.3472 (2)	0.3471 (3)	0.9197 (3)	0.0519 (9)	
H37	0.3027	0.3443	0.9651	0.062*	
N1	0.10891 (14)	0.57350 (18)	0.4841 (2)	0.0330 (6)	
N2	0.14345 (15)	0.17067 (18)	0.5281 (3)	0.0398 (6)	
H2A	0.1097	0.1391	0.4684	0.048*	
H2B	0.1741	0.1389	0.5917	0.048*	
N3	0.25616 (14)	0.53850 (17)	0.7866 (2)	0.0342 (6)	
H3	0.2651	0.5665	0.8644	0.041*	
N4	0.09609 (13)	0.31051 (18)	0.4158 (2)	0.0326 (6)	
N5	0.02048 (14)	0.42620 (19)	0.3037 (2)	0.0374 (6)	
H5	0.0021	0.4842	0.2801	0.045*	
O1	0.05174 (13)	0.60506 (16)	0.3938 (2)	0.0445 (6)	
O2	0.14848 (13)	0.63220 (15)	0.5658 (2)	0.0431 (6)	
O3	0.13161 (12)	0.46735 (14)	0.80020 (19)	0.0352 (5)	
O4	0.24819 (17)	0.18945 (18)	0.7443 (3)	0.0682 (8)	
O5	0.28997 (18)	0.6227 (3)	0.0417 (3)	0.0927 (12)	
S1	0.24911 (7)	0.59246 (8)	0.15647 (10)	0.0650 (3)	

### Atomic displacement parameters (Å^2^)

**Table:**

	*U*^11^	*U*^22^	*U*^33^	*U*^12^	*U*^13^	*U*^23^
C1	0.0223 (13)	0.0279 (15)	0.0298 (14)	0.0015 (11)	−0.0003 (11)	0.0011 (12)
C2	0.0203 (13)	0.0296 (14)	0.0287 (14)	−0.0009 (11)	−0.0009 (11)	−0.0003 (12)
C3	0.0221 (13)	0.0311 (15)	0.0312 (15)	−0.0011 (11)	0.0015 (11)	−0.0002 (12)
C4	0.0213 (13)	0.0322 (16)	0.0343 (15)	−0.0013 (11)	0.0048 (11)	−0.0007 (12)
C5	0.0209 (13)	0.0380 (17)	0.0283 (14)	0.0006 (11)	0.0034 (11)	0.0006 (12)
C6	0.0273 (16)	0.057 (2)	0.0399 (17)	0.0011 (14)	−0.0074 (13)	−0.0116 (15)
C7	0.0305 (16)	0.0461 (19)	0.0474 (19)	−0.0066 (14)	−0.0093 (14)	−0.0115 (15)
C8	0.0247 (14)	0.0260 (14)	0.0352 (15)	0.0037 (11)	−0.0007 (12)	0.0025 (12)
C9	0.0234 (14)	0.0331 (16)	0.0404 (16)	0.0002 (12)	−0.0006 (12)	−0.0012 (13)
C10	0.0192 (13)	0.0307 (15)	0.0366 (15)	0.0018 (11)	−0.0009 (11)	0.0020 (12)
C11	0.0277 (15)	0.0436 (18)	0.0385 (17)	0.0032 (13)	0.0015 (12)	0.0028 (14)
C12	0.0321 (17)	0.054 (2)	0.058 (2)	−0.0006 (15)	0.0134 (15)	0.0095 (17)
C13	0.0248 (16)	0.050 (2)	0.073 (2)	−0.0077 (14)	0.0066 (16)	0.0033 (18)
C14	0.0257 (15)	0.0425 (19)	0.058 (2)	−0.0073 (13)	−0.0031 (14)	−0.0093 (16)
C15	0.074 (3)	0.059 (2)	0.049 (2)	0.005 (2)	0.0023 (19)	−0.0067 (18)
C16	0.059 (3)	0.089 (3)	0.071 (3)	−0.006 (2)	0.012 (2)	−0.018 (2)
C31	0.0331 (16)	0.0346 (17)	0.0383 (17)	0.0008 (13)	−0.0012 (13)	0.0013 (13)
C32	0.0307 (16)	0.0320 (16)	0.0423 (17)	0.0042 (12)	−0.0049 (13)	0.0066 (13)
C33	0.0407 (19)	0.050 (2)	0.058 (2)	0.0070 (15)	0.0094 (16)	0.0066 (17)
C34	0.0297 (19)	0.068 (3)	0.103 (4)	0.0031 (18)	0.007 (2)	0.021 (2)
C35	0.042 (2)	0.081 (3)	0.095 (3)	−0.020 (2)	−0.024 (2)	0.028 (3)
C36	0.073 (3)	0.081 (3)	0.050 (2)	−0.023 (2)	−0.021 (2)	0.009 (2)
C37	0.047 (2)	0.064 (2)	0.0402 (19)	−0.0104 (17)	−0.0042 (15)	0.0026 (17)
N1	0.0254 (12)	0.0367 (14)	0.0352 (13)	0.0021 (10)	0.0006 (10)	0.0050 (11)
N2	0.0381 (14)	0.0295 (14)	0.0482 (15)	−0.0054 (11)	−0.0028 (12)	−0.0016 (11)
N3	0.0299 (13)	0.0379 (14)	0.0322 (13)	−0.0008 (10)	−0.0019 (10)	−0.0067 (11)
N4	0.0218 (12)	0.0362 (14)	0.0364 (13)	0.0002 (10)	−0.0045 (10)	−0.0058 (11)
N5	0.0274 (13)	0.0447 (15)	0.0355 (13)	0.0032 (11)	−0.0080 (10)	−0.0004 (12)
O1	0.0371 (12)	0.0430 (13)	0.0466 (13)	0.0088 (10)	−0.0117 (10)	0.0091 (10)
O2	0.0402 (12)	0.0334 (12)	0.0500 (13)	−0.0012 (9)	−0.0083 (10)	−0.0052 (10)
O3	0.0287 (11)	0.0408 (12)	0.0360 (11)	0.0024 (9)	0.0054 (9)	0.0033 (9)
O4	0.0734 (18)	0.0428 (15)	0.0733 (18)	−0.0118 (13)	−0.0298 (14)	0.0201 (13)
O5	0.0581 (18)	0.163 (3)	0.0610 (17)	−0.0245 (19)	0.0210 (14)	−0.053 (2)
S1	0.0735 (7)	0.0610 (7)	0.0544 (6)	0.0189 (5)	−0.0064 (5)	−0.0117 (5)

### Geometric parameters (Å, º)

**Table:**

C1—N1	1.380 (4)	C13—H13	0.9300
C1—C5	1.384 (4)	C14—H14	0.9300
C1—C2	1.514 (3)	C15—S1	1.769 (4)
C2—C10	1.515 (4)	C15—H15A	0.9600
C2—C3	1.542 (4)	C15—H15B	0.9600
C2—C8	1.558 (4)	C15—H15C	0.9600
C3—C4	1.383 (4)	C16—S1	1.771 (4)
C3—C31	1.439 (4)	C16—H16A	0.9600
C4—N2	1.340 (4)	C16—H16B	0.9600
C4—N4	1.370 (3)	C16—H16C	0.9600
C5—N5	1.324 (3)	C31—O4	1.242 (4)
C5—N4	1.357 (4)	C31—C32	1.504 (4)
C6—N5	1.454 (4)	C32—C37	1.380 (5)
C6—C7	1.509 (4)	C32—C33	1.385 (4)
C6—H6A	0.9700	C33—C34	1.379 (5)
C6—H6B	0.9700	C33—H33	0.9300
C7—N4	1.468 (3)	C34—C35	1.364 (6)
C7—H7A	0.9700	C34—H34	0.9300
C7—H7B	0.9700	C35—C36	1.372 (6)
C8—O3	1.232 (3)	C35—H35	0.9300
C8—N3	1.337 (4)	C36—C37	1.382 (5)
C9—C14	1.373 (4)	C36—H36	0.9300
C9—C10	1.385 (4)	C37—H37	0.9300
C9—N3	1.401 (4)	N1—O2	1.245 (3)
C10—C11	1.375 (4)	N1—O1	1.266 (3)
C11—C12	1.386 (4)	N2—H2A	0.8600
C11—H11	0.9300	N2—H2B	0.8600
C12—C13	1.380 (5)	N3—H3	0.8600
C12—H12	0.9300	N5—H5	0.8600
C13—C14	1.384 (5)	O5—S1	1.491 (3)
			
N1—C1—C5	118.8 (2)	S1—C15—H15A	109.5
N1—C1—C2	118.4 (2)	S1—C15—H15B	109.5
C5—C1—C2	122.8 (2)	H15A—C15—H15B	109.5
C1—C2—C10	112.2 (2)	S1—C15—H15C	109.5
C1—C2—C3	110.7 (2)	H15A—C15—H15C	109.5
C10—C2—C3	114.4 (2)	H15B—C15—H15C	109.5
C1—C2—C8	110.2 (2)	S1—C16—H16A	109.5
C10—C2—C8	100.5 (2)	S1—C16—H16B	109.5
C3—C2—C8	108.2 (2)	H16A—C16—H16B	109.5
C4—C3—C31	117.4 (3)	S1—C16—H16C	109.5
C4—C3—C2	120.7 (2)	H16A—C16—H16C	109.5
C31—C3—C2	121.9 (2)	H16B—C16—H16C	109.5
N2—C4—N4	114.0 (2)	O4—C31—C3	121.9 (3)
N2—C4—C3	124.7 (3)	O4—C31—C32	113.9 (3)
N4—C4—C3	121.4 (2)	C3—C31—C32	124.1 (3)
N5—C5—N4	109.5 (2)	C37—C32—C33	119.9 (3)
N5—C5—C1	130.4 (3)	C37—C32—C31	121.4 (3)
N4—C5—C1	120.1 (2)	C33—C32—C31	118.1 (3)
N5—C6—C7	103.8 (2)	C34—C33—C32	119.3 (4)
N5—C6—H6A	111.0	C34—C33—H33	120.3
C7—C6—H6A	111.0	C32—C33—H33	120.3
N5—C6—H6B	111.0	C35—C34—C33	120.8 (4)
C7—C6—H6B	111.0	C35—C34—H34	119.6
H6A—C6—H6B	109.0	C33—C34—H34	119.6
N4—C7—C6	103.1 (2)	C34—C35—C36	120.1 (4)
N4—C7—H7A	111.2	C34—C35—H35	120.0
C6—C7—H7A	111.2	C36—C35—H35	120.0
N4—C7—H7B	111.2	C35—C36—C37	120.1 (4)
C6—C7—H7B	111.2	C35—C36—H36	120.0
H7A—C7—H7B	109.1	C37—C36—H36	120.0
O3—C8—N3	126.2 (3)	C32—C37—C36	119.8 (4)
O3—C8—C2	125.1 (2)	C32—C37—H37	120.1
N3—C8—C2	108.7 (2)	C36—C37—H37	120.1
C14—C9—C10	121.9 (3)	O2—N1—O1	120.1 (2)
C14—C9—N3	128.6 (3)	O2—N1—C1	119.3 (2)
C10—C9—N3	109.5 (2)	O1—N1—C1	120.6 (2)
C11—C10—C9	120.5 (3)	C4—N2—H2A	120.0
C11—C10—C2	130.2 (3)	C4—N2—H2B	120.0
C9—C10—C2	109.3 (2)	H2A—N2—H2B	120.0
C10—C11—C12	118.1 (3)	C8—N3—C9	111.9 (2)
C10—C11—H11	120.9	C8—N3—H3	124.1
C12—C11—H11	120.9	C9—N3—H3	124.1
C13—C12—C11	120.8 (3)	C5—N4—C4	122.7 (2)
C13—C12—H12	119.6	C5—N4—C7	111.1 (2)
C11—C12—H12	119.6	C4—N4—C7	124.9 (2)
C12—C13—C14	121.3 (3)	C5—N5—C6	112.4 (2)
C12—C13—H13	119.3	C5—N5—H5	123.8
C14—C13—H13	119.3	C6—N5—H5	123.8
C9—C14—C13	117.3 (3)	O5—S1—C15	106.5 (2)
C9—C14—H14	121.3	O5—S1—C16	105.0 (2)
C13—C14—H14	121.3	C15—S1—C16	97.2 (2)
			
N1—C1—C2—C10	−60.0 (3)	C10—C9—C14—C13	−0.1 (5)
C5—C1—C2—C10	118.2 (3)	N3—C9—C14—C13	179.9 (3)
N1—C1—C2—C3	170.8 (2)	C12—C13—C14—C9	−0.4 (5)
C5—C1—C2—C3	−10.9 (4)	C4—C3—C31—O4	−18.9 (5)
N1—C1—C2—C8	51.1 (3)	C2—C3—C31—O4	162.4 (3)
C5—C1—C2—C8	−130.6 (3)	C4—C3—C31—C32	157.3 (3)
C1—C2—C3—C4	6.8 (4)	C2—C3—C31—C32	−21.4 (4)
C10—C2—C3—C4	−121.2 (3)	O4—C31—C32—C37	−73.6 (4)
C8—C2—C3—C4	127.7 (3)	C3—C31—C32—C37	109.9 (4)
C1—C2—C3—C31	−174.6 (2)	O4—C31—C32—C33	96.9 (4)
C10—C2—C3—C31	57.4 (3)	C3—C31—C32—C33	−79.6 (4)
C8—C2—C3—C31	−53.7 (3)	C37—C32—C33—C34	0.2 (5)
C31—C3—C4—N2	4.5 (4)	C31—C32—C33—C34	−170.4 (3)
C2—C3—C4—N2	−176.8 (3)	C32—C33—C34—C35	−1.3 (6)
C31—C3—C4—N4	−174.9 (3)	C33—C34—C35—C36	0.3 (6)
C2—C3—C4—N4	3.8 (4)	C34—C35—C36—C37	1.8 (7)
N1—C1—C5—N5	1.4 (5)	C33—C32—C37—C36	1.9 (5)
C2—C1—C5—N5	−176.8 (3)	C31—C32—C37—C36	172.2 (3)
N1—C1—C5—N4	−177.4 (2)	C35—C36—C37—C32	−2.8 (6)
C2—C1—C5—N4	4.4 (4)	C5—C1—N1—O2	178.4 (2)
N5—C6—C7—N4	−4.7 (3)	C2—C1—N1—O2	−3.3 (4)
C1—C2—C8—O3	61.2 (3)	C5—C1—N1—O1	−0.7 (4)
C10—C2—C8—O3	179.8 (3)	C2—C1—N1—O1	177.6 (2)
C3—C2—C8—O3	−60.0 (3)	O3—C8—N3—C9	−179.0 (3)
C1—C2—C8—N3	−121.4 (2)	C2—C8—N3—C9	3.6 (3)
C10—C2—C8—N3	−2.8 (3)	C14—C9—N3—C8	177.1 (3)
C3—C2—C8—N3	117.4 (2)	C10—C9—N3—C8	−2.9 (3)
C14—C9—C10—C11	0.8 (4)	N5—C5—N4—C4	−171.2 (2)
N3—C9—C10—C11	−179.2 (2)	C1—C5—N4—C4	7.8 (4)
C14—C9—C10—C2	−179.2 (3)	N5—C5—N4—C7	−3.7 (3)
N3—C9—C10—C2	0.9 (3)	C1—C5—N4—C7	175.3 (3)
C1—C2—C10—C11	−61.7 (4)	N2—C4—N4—C5	168.5 (3)
C3—C2—C10—C11	65.5 (4)	C3—C4—N4—C5	−12.0 (4)
C8—C2—C10—C11	−178.8 (3)	N2—C4—N4—C7	2.8 (4)
C1—C2—C10—C9	118.2 (3)	C3—C4—N4—C7	−177.8 (3)
C3—C2—C10—C9	−114.6 (3)	C6—C7—N4—C5	5.3 (3)
C8—C2—C10—C9	1.1 (3)	C6—C7—N4—C4	172.5 (3)
C9—C10—C11—C12	−0.9 (4)	N4—C5—N5—C6	0.3 (3)
C2—C10—C11—C12	179.0 (3)	C1—C5—N5—C6	−178.6 (3)
C10—C11—C12—C13	0.4 (5)	C7—C6—N5—C5	3.0 (3)
C11—C12—C13—C14	0.3 (5)		

### Hydrogen-bond geometry (Å, º)

**Table:**

*D*—H···*A*	*D*—H	H···*A*	*D*···*A*	*D*—H···*A*
N2—H2*B*···O4	0.86	1.92	2.549 (3)	129
N2—H2*A*···O3^i^	0.86	2.30	2.939 (3)	131
N3—H3···O5^ii^	0.86	1.92	2.779 (4)	177
N5—H5···O1	0.86	2.08	2.604 (3)	119
N5—H5···O3^iii^	0.86	2.31	2.924 (3)	129
C6—H6*B*···O2^iii^	0.97	2.59	3.274 (4)	128
C11—H11···O4^i^	0.93	2.43	3.346 (4)	167
C15—H15*C*···O2^iv^	0.96	2.55	3.448 (4)	156

Symmetry codes: (i) *x*, −*y*+1/2, *z*−1/2; (ii) *x*, *y*, *z*+1; (iii) −*x*, −*y*+1, −*z*+1; (iv) *x*, −*y*+3/2, *z*−1/2.
